# Gene Expression Profiling of Early Hepatic Stellate Cell Activation Reveals a Role for Igfbp3 in Cell Migration

**DOI:** 10.1371/journal.pone.0084071

**Published:** 2013-12-17

**Authors:** Inge Mannaerts, Ben Schroyen, Stefaan Verhulst, Leentje Van Lommel, Frans Schuit, Marc Nyssen, Leo A. van Grunsven

**Affiliations:** 1 Liver Cell Biology Laboratory, Vrije Universiteit Brussel, Brussel, Belgium; 2 Department of Cellular and Molecular Medicine, Leuven, Belgium; 3 Department of Biostatistics and Medical Informatics, Faculty of Medicine and Pharmacy,Vrije Universiteit Brussel, Brussel, Belgium; Institute of Hepatology, Foundation for Liver Research, United Kingdom

## Abstract

**Background:**

Scarring of the liver is the result of prolonged exposure to exogenous or endogenous stimuli. At the onset of fibrosis, quiescent hepatic stellate cells (HSCs) activate and transdifferentiate into matrix producing, myofibroblast-like cells.

**Aim and methods:**

To identify key players during early HSC activation, gene expression profiling was performed on primary mouse HSCs cultured for 4, 16 and 64 hours. Since valproic acid (VPA) can partly inhibit HSC activation, we included VPA-treated cells in the profiling experiments to facilitate this search.

**Results:**

Gene expression profiling confirmed early changes for known genes related to HSC activation such as *alpha* *smooth* *muscle* *actin* (*Acta2*)*, lysyl* *oxidase* (*Lox*) and *collagen*, type *I*, alpha *1* (*Col1a1*). In addition we noticed that, although genes which are related to fibrosis change between 4 and 16 hours in culture, most gene expression changes occur between 16 and 64 hours. *Insulin-like* *growth* *factor* *binding* protein *3* (*Igfbp3*) was identified as a gene strongly affected by VPA treatment. During normal HSC activation *Igfbp3* is up regulated and this can thus be prevented by VPA treatment *in* *vitro* and *in* *vivo*. siRNA-mediated silencing of *Igfbp3* in primary mouse HSCs induced matrix metalloproteinase (Mmp) *9* mRNA expression and strongly reduced cell migration. The reduced cell migration after *Igfbp3* knock-down could be overcome by tissue inhibitor of metalloproteinase (TIMP) 1 treatment.

**Conclusion:**

Igfbp3 is a marker for culture-activated HSCs and plays a role in HSC migration. VPA treatment prevents *Igfbp3* transcription during activation of HSCs *in* *vitro* and *in* *vivo*.

## Introduction

Hepatic stellate cell (HSC) activation was identified many years ago as a key event in the development of liver fibrosis [1]. In normal livers, HSCs are in a quiescent state and are characterized by the presence of lipid droplets, long cytoplasmic processes and a balanced production of matrix proteins and matrix remodeling enzymes to maintain an optimal environment for the resident liver cells [2,3]. When the liver is damaged, HSCs become activated, a process stimulated by the presence of inflammatory cytokines and resulting in a myofibroblast-like phenotype [4]. Upon activation, HSCs loose the expression of quiescent markers like neurotrimin and plexin C1 [5], they express high levels of alpha smooth muscle actin (α-SMA), produce large amounts of fibrillar collagens and gain migratory and contractile properties [6,7]. Sustained stellate cell activation results in disturbed liver architecture and function. Recent studies in mice show the reversibility of the activated stellate cells to quiescent HSCs during the reversal of experimental fibrosis [8,9]. Therefore, studying the activation process of HSCs is interesting for the development of anti-fibrotic therapies. Gene expression data are available for *in vitro* activated HSCs and for HSCs isolated from different animal injury models [5,10–12]. However, the profiling was always performed after at least 24 hours of culture. We hypothesized that gene expression profiling during the earliest time points in culture could identify key genes involved in the onset of HSC activation. From previous studies we know that histone deacetylase (HDAC) inhibitors, like valproic acid (VPA), can maintain these cells in a more quiescent state [13] and reduce fibrogenesis in animal models for liver, kidney and heart fibrosis [14]. HDACs are enzymes involved in chromatin remodeling and in gene expression alterations [15]. In this study we chose to compare the gene expression profiles of *in vitro* cultured cells in normal conditions to those treated with VPA, in order to identify genes involved in the earliest stages of the activation of freshly isolated mouse HSCs.

Our analyses identified 1274 genes that were significantly changed in HSCs between 4 and 64 hours in culture. Of these differentially expressed genes, 147 genes were normalized by VPA-treatment during culture. Expression changes of a selected set of genes were confirmed in culture activated HSCs and in cells isolated from mice treated with carbon tetrachloride (CCl_4_) ± VPA. While no new key regulators of HSC activation *in vivo* could be identified using this approach, we did establish Insulin-like growth factor binding protein 3 (IGFBP3) as an early HSC activation marker that can be regulated by VPA. We therefore further investigated the role of this protein by siRNA-mediated knock down in freshly isolated mouse HSCs which revealed a function for IGFBP3 in HSC migration.

## Materials and Methods

### Isolation and culturing of mouse cells

All procedures on animals were carried out in accordance with University's guidelines for the care and use of laboratory animals in research, the performed experiments were approved by the ethical committee of the Vrije Universiteit Brussel in project 10-212-3. Balb/cByJ mice (25-35g) were purchased from Charles River Laboratories (L’Arbresle, France). The mice were housed in a controlled environment with free access to chow and water. Prior to surgery, the mice were anesthetised with Nembutal (CEVA Santé Animale, Brussels, Belgium) according to their body weight. HSCs, liver sinusoidal endothelial cells (LSECs) and hepatocytes were isolated, cultured and characterised as previously described [13,16]. Kupffer cells (KC) were isolated from the non-parenchymal fraction after after the collagenase/pronase perfusion by fluorescence-activated cell sorting (FACS) using an F4/80 antibody (Invitrogen, Eugene, OR). Cell purity was checked by morphology and qPCR analysis for marker genes (Cyp3a11 (HEP), CD32b (LSEC), F4/80 (KC), Desmin, Gfap and Acta2 (HSCs)) as published by Schroyen et al [16]. Based on differences in dCt values for the different cell type markers in the sorted populations the percentage of contaminating cells in our populations ranges from 0.4 to 1.5%.

Mice that were used for the isolation of *in vivo*-activated HSCs underwent four intraperitoneal injections over two weeks’ time of 50 µL CCl_4_/100g body weight in mineral oil (Sigma-Aldrich, St. Louis, MO) [5]. To study the effect of VPA on *in vivo* HSC activation, mice received drinking water containing 0,4% VPA twice a week, starting two days before the first CCl_4_ injection [17].

### RNA extraction, reverse-transcription and PCR

Total RNA was extracted and purified from cultured cells using the ReliaPrep RNA Cell Miniprep System (Promega, Madison, WI). Total RNA was converted to cDNA by reverse-transcription using the Revert Aid Kit (ThermoFisher Scientific, St. Leon-Rot, Germany). The RT reaction was performed at 25 °C for ten minutes followed by 30 minutes at 50 °C. For quantitative real-time polymerase chain reaction (qPCR), GoTaq QPCR Master Mix with BRYTE green (Promega, Madison, WI) was used, subjected to qPCR in a 7500 real time PCR system and analysed using System SDS software v2.0.5 (Applied Biosystems) using GAPDH for normalisation whose expression in HSCs is not affected by treatments nor by cell culture (data not shown). Fold change differences between samples were determined using the comparative Ct (δδCt) method. The expression level of different targets, relative to GAPDH and relative to the calibrator, was given by 2^-δδCt^. Gene-specific primers produced by Integrated DNA Technologies (Leuven, Belgium) are listed in Table 1.

**Table 1:** Overview of the primer combinations for the different genes.

**Target gene**	**Accession**	**Forward primer (5’-3’)**	**Reverse primer (5’-3’)**
***Gapdh***	NM_008084	tgtccgtcgtggatctgac	cctgcttcaccaccttcttg
***Acta2***	NM_007392	ccagcaccatgaagatcaag	tggaaggtagacagcgaagc
***Col1a1***	NM_007742	acctaagggtaccgctgga	tccagcttctccatctttgc
***Lox***	NM_010728	ctcctgggagtggcacag	cttgctttgtggccttcag
***Desmin***	NM_010043	gccacctaccggaagctact	gcagagaaggtctggataggaa
***Igfbp3***	NM_008343	gcagcctaagcacctacctc	tcctcctcggactcactgat
***Serpina1b***	NM_009244	caatggggctgacctctct	gcacagccttatgcacagc
***Serpina1e***	NM_009247	ggctgacctctccggaat	gtcagcacggccttatgc
***Cyp7b1***	NM_007825	ggagccacgaccctagatg	gccatgccaagataaggaagc
***Tmem204***	NM_001001183	tccctcatcctcaacaacgc	gactcccttacccctgtcca
***Plat***	NM_008872	tgaccagggaatacatgggag	gtctgcgttggctcatctctg
***Sort***	NM_001271599	cccggacttcatcgccaag	aggacgagaataaccccagtg
***Uchl***	NM_011670	agggacaggaagttagcccta	agcttctccgtttcagacaga
***Ptprn***	NM_008985	tgtttgaccgcagactttgtt	ggagcacaccttgtaggcg
***Aplp1***	NM_007467	gtgggcgtctaacccttcac	ctgcgggtccagaagacag
***Vcl***	NM_009502.4	cctcaggagcctgacttcc	agccagctcatcagttagtcg
***Pxn***	NM_011223.2	tactggagctgaacgcagtg	ccaagggagtgttattttctgg
***Myh9***	NM_022410	acaatggaggccatgagaat	gagatgacccgcagcaag
***Myh10***	NM_175260	ggaggacaccctagacacca	ccacttcctgctcacgtttt
***Myh11Common assay for 2 isoforms***	NM_001161775.1 & NM_013607.2	gaggagcaggttgaacagga	gcttcttgtccttttgcttca
***Mmp1a***	NM_032006	aactacatttaggggagaggtgt	gcagcgtcaagtttaactggaa
***Mmp2***	NM_008610	aactttgagaaggatggcaagt	tgccacccatggtaaacaa
***Mmp3***	NM_010809	acatggagactttgtcccttttg	ttggctgagtggtagagtccc
***Mmp9***	NM_013599	ctggacagccagacactaaag	ctcgcggcaagtcttcagag
***Mmp11***	NM_008606	ccggagagtcaccgtcatc	gcaggactagggacccaatg
***Mmp13***	NM_008607	tgtttgcagagcactacttgaa	cagtcacctctaagccaaagaaa
***Mmp19 tv1***	NM_021412	ctgtggctggcattcttactt	gggcagtccagatgcttcc
***Mmp19 tv2***	NM_001164197	atggatgacgccacaaggg	cacctcccggaaggtcaga

TV: Transcript variant.

### Gene expression profiling and analysis

Double-stranded cDNA was synthesised from total RNA originating from cultured mouse HSCs. After *in vitro* transcription and fragmentation, cRNA was biotin labelled, 2 µg of the labelled cRNA was hybridised to Affymetrix GeneChip Mouse Gene 1.0 ST arrays (Affymetrix, Santa Clara, CA). Three independent biological replicates were included in each of the groups: control 4 hours (Ctrl4h), control 64 hours (Ctrl64h), VPA 4 hours (VPA4h) and VPA 64 hours (VPA64h), only for the Ctrl16h sample just two samples were taken. The hybridisation cocktail, including the fragmented target and probe array controls, was then hybridised to the probe arrays during a 16-hour incubation period. Next, the arrays underwent automated washing and staining on a fluidics station and intensities were measured by a scanner. The *.cel files were imported into GeneSpringGX 11 (Agilent, Santa Clara, CA) for normalisation and data analysis. To create an expression matrix, the raw data were pre-processed using the robust multi-array average (RMA) algorithm consisting of background correction, quantile normalisation & probe summarisation. Sample and hybridisation quality was checked by principal component analysis (PCA) and by analysing the hybridisation controls. A fold change cut-off of 2.0 in combination with an ANOVA identified differentially expressed transcripts. p-Value computation was done asymptotically (p-value cut-off of 0.05) and Benjamini-Hochberg was used for multiple testing corrections. Potential VPA target genes were identified by using a clustering algorithm on transcripts that were at least two-fold differentially expressed between Ctrl64h and VPA64h. Raw data are made publically available on the NCBI Gene Expression Omnibus database, with accession number GSE51882.

### Small Interfering RNA transfection

Predesigned double-stranded small interfering RNAs (siRNAs) from Integrated DNA Technologies were used; Igfbp3 siRNA 1 (MMC.RNAI.N008343.12.1) & Igfbp3 siRNA 2 (MMC.RNAI.N008343.12.2). This siRNA-mix (Igfbp3 siRNA1 + siRNA2, 5 nM) was transfected using HiPerfect Transfection Reagent (Qiagen) according to the manufacturer’s instructions. Cells were either transfected once (at day 1, for EdU proliferation assay) or twice during HSC activation (at day 5 and day 7, for migration assays). A non-silencing siRNA from Integrated DNA Technologies was used as a control.

### Western blot

Cells were washed with ice-cold phosphate-buffered saline, scraped and incubated for 10 min on a rotator (SB2, Stuart, Staffordshire, UK) at 4 °C with lysis buffer (170 mM NaCl, 10 mM EDTA, 50 mM Tris pH 7.4, 50 mM NaF, 0.2 mM dithiothreitol and 0.5% NP-40) supplemented with protease and phosphatase inhibitors. The protein concentration was measured using a bicinchoninic acid (BCA) determination kit (Pierce Chemical Co, Rockford, IL). Twenty micrograms of protein were separated on a 8% Tris–glycine SDS-Polyacrylamide gel and transferred onto polyvinyldifluoride (PVDF) membranes (Amersham Biosciences, Little Chalfont, UK) using a wet blotting apparatus (Mini Trans-Blot Cell, BioRad, Nazareth, Belgium). Afterwards the membrane was blocked by 5% milk in PBS-Tween. After overnight incubation with the primary MMP9 antibody (IM10L, Oncogene Science, Cambridge, MA) and one hour incubation with a horseradish peroxidase conjugated secondary antibody (1/30000) (Dako, Glostrup, Denmark), proteins were visualized with an ECL chemiluminescence detection system (Pierce Chemical Co.).

### Cell proliferation assay

Cell proliferation was measured as active DNA synthesis with the Click-iT EdU Cell Proliferation Assay Kit (Invitrogen, Eugene, OR). HSCs were plated and transfected with a non-silencing RNA (siCtrl) or an siRNA-mix for Igfbp3 (siIgfbp3) at day one post isolation. 48 Hours later (day three) EdU labeling was started. At day five, cells were formalin fixed and visualization of the EdU incorporation was obtained according to the manufacturer’s instructions. The ratio of EdU positive cells on total cells and was calculated.

### Migration assays

To investigate the effect of siIgfbp3 on HSC migration, two different assays were used: a wound healing assay and a transwell (Boyden chamber) assay.

For the wound-induced migration assays, nine-day-old HSCs, that were transfected at day five and day seven with a siRNA-mix for Igfbp3, were seeded in 6 cm polystyrene culture dishes. 24-Hours later, when the attached cells were confluent, serum-free DMEM with or without 100 ng/mL recombinant murine tissue inhibitor of matrix metalloproteinase-1 (TIMP-1, R&D systems, Minneapolis, MN) or MMP9 selective inhibitor (MMP9inh, Calbiochem, Merck, Darmstadt, Germany) was added to the plate and a ‘wound’ was made with a micropipette tip. Pictures were taken (10X magnification) at zero and 48 hours post ‘wounding’ using an Axiovert 100 microscope and AxioCam MRc 5 digital camera (Carl Zeiss, Zaventem, Belgium). Cell migration was analyzed by quantifying the size of the wound area (number of pixels) using the free image processing and analysis software ImageJ (http://imagej.nih.gov/ij/index.html).

For the transwell assays, nine-day-old HSCs, that were transfected with a siRNA-mix for Igfbp3, were seeded in collagen-coated Boyden chambers residing in a 24-well plate with serum-free DMEM. After 60 minutes the chambers were transferred to new wells with serum-free medium containing 20 ng/mL platelet derived growth factor (PDGFbb). After 18 hours, cells were removed from the top of the transwell inserts and were washed with PBS, fixed for one minute with ice-cold 100% methanol and mounted with Prolong Gold antifade reagent with DAPI (Invitrogen). PDGF-induced cell migration was analyzed by counting the number of nuclei (= cells) on the lower membrane using fluorescence microscopy (Carl Zeiss). 

### Immunocytochemistry

After 64 hours of culture, in the presence or absence of 2.5 mM VPA, HSCs were washed with PBS and fixed for 10 minutes with 4% buffered formaldehyde pH 6.9 (Merck, Darmstadt, Germany). Following permeabilization with 0.1% Triton-X 100 (in PBS with 1% bovine serum albumin), cells were incubated overnight with the primary antibody (α-SMA, 1/1000 [Sigma]). Antibody binding was visualized using Alexa488-labeled secondary antibody (1/200). Images were taken with an AxioCam MRc 5 digital camera (Carl Zeiss).

### Statistical analysis

GraphPad Prism v6.0.1 (GraphPad Software, La Jolla, CA) was used for statistical analysis. Data in the figures are expressed as means ± SEM. Differences among groups were tested for statistical signiﬁcance by Student *t*-test or analysis of variance (ANOVA) followed by Bonferroni, depending on the number of groups. *ns* = not significant p ≥ 0.05, * p < 0.05, ** p < 0.01, *** p < 0,001.

## Results

### Valproic acid prevents changes during early stellate cell activation

**Figure 1:**
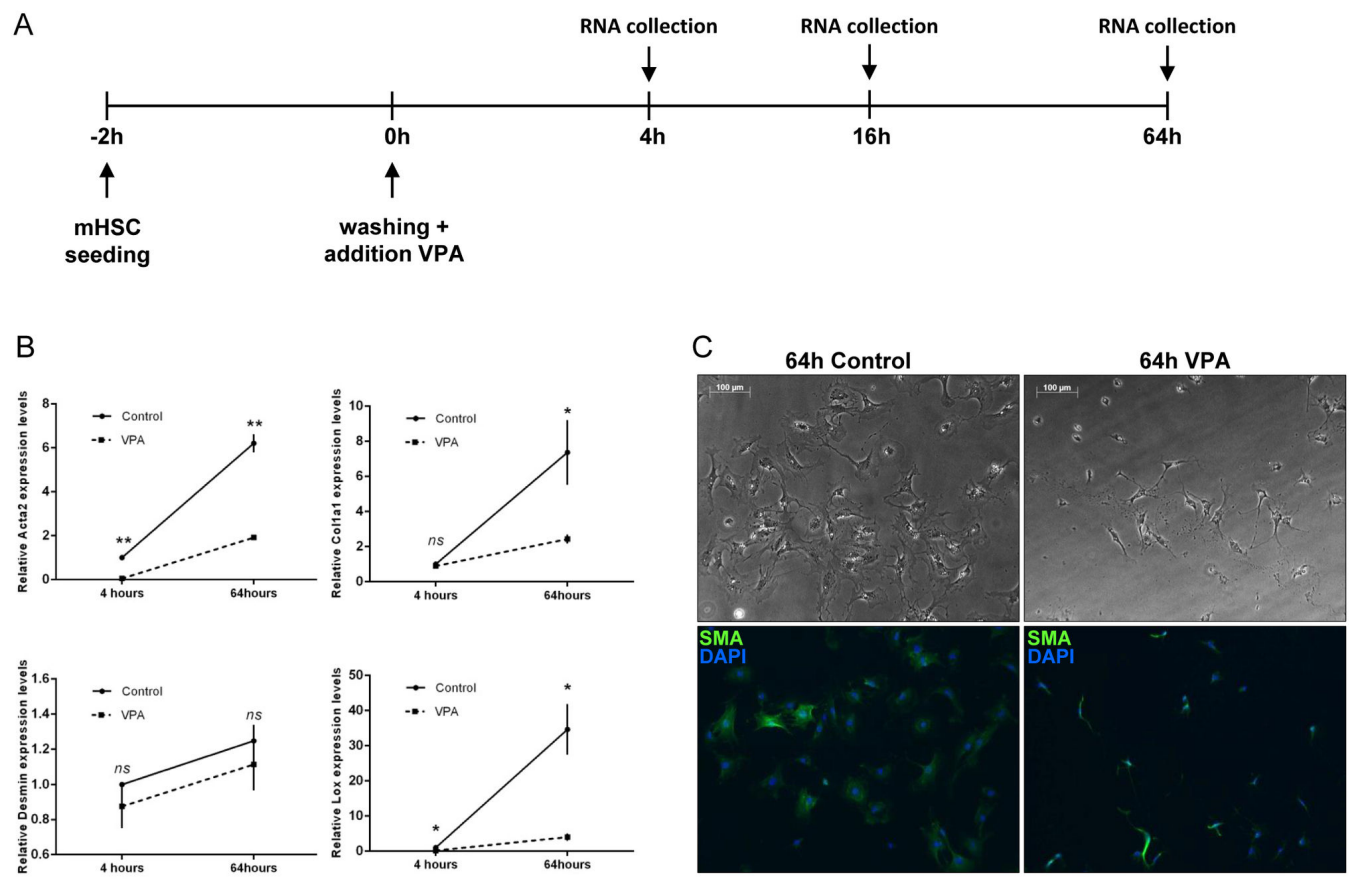
Experimental set-up and sample validation. (**A**) Mouse hepatic stellate cells (HSCs) were isolated from Balb/C CBY jicco mouse livers by Nycodenz gradient. Next, the cells were cultured for 2 hours after which unattached cells and debris were removed by washing. The HSCs were further cultured for 4, 16 and 64 hours in the presence or absence of valproic acid (VPA). (**B**) At the indicated time points, cells were collected for mRNA analysis of HSC activation genes actin, alpha *2*, smooth muscle, aorta (Acta2), lysyl oxidase (Lox), collagen, type *I*, alpha *1* (Col1a1) and Desmin. (**C**) In addition, immunocytochemistry for α-smooth muscle actin (SMA) was performed on cells cultured for 64 hours. Staining was visualized using a secondary Alexa-488 antibody and nuclei were stained with 4’,6-Diamidino-2-phenylindole (DAPI). Experiments were repeated at least 3 times. In the graphs, the results are displayed as means ± SEM. ns = not significant p ≥ 0.05, * p < 0.05, ** p < 0.01.

HSCs were isolated from healthy Balb/cByJ mice and cultured for 4, 16 and 64 hours after washing, either in the presence or absence of VPA (Figure 1A). QPCR analysis for mRNA levels of activation markers *Acta2, Col1a1* and *Lox*, encoding for respectively α-smooth muscle actin, collagen 1a1 and lysyl oxidase, confirmed our previous observations that VPA treatment can prevent the activation of HSCs, not only when cultured for days as shown before by Mannaerts et al [13], but even at very early time points (Figure 1B). This was also shown by immunocytochemistry for α-SMA, showing clear positivity already in 64 hours cultured HSCs while α-SMA positivity was almost absent in cells treated with VPA (Figure 1C). After validation of the sample quality and confirmation of the VPA effectiveness, we performed gene expression profiling by microarrays in order to identify early regulators of HSC activation.

### Early gene expression changes during hepatic stellate cell activation

**Figure 2:**
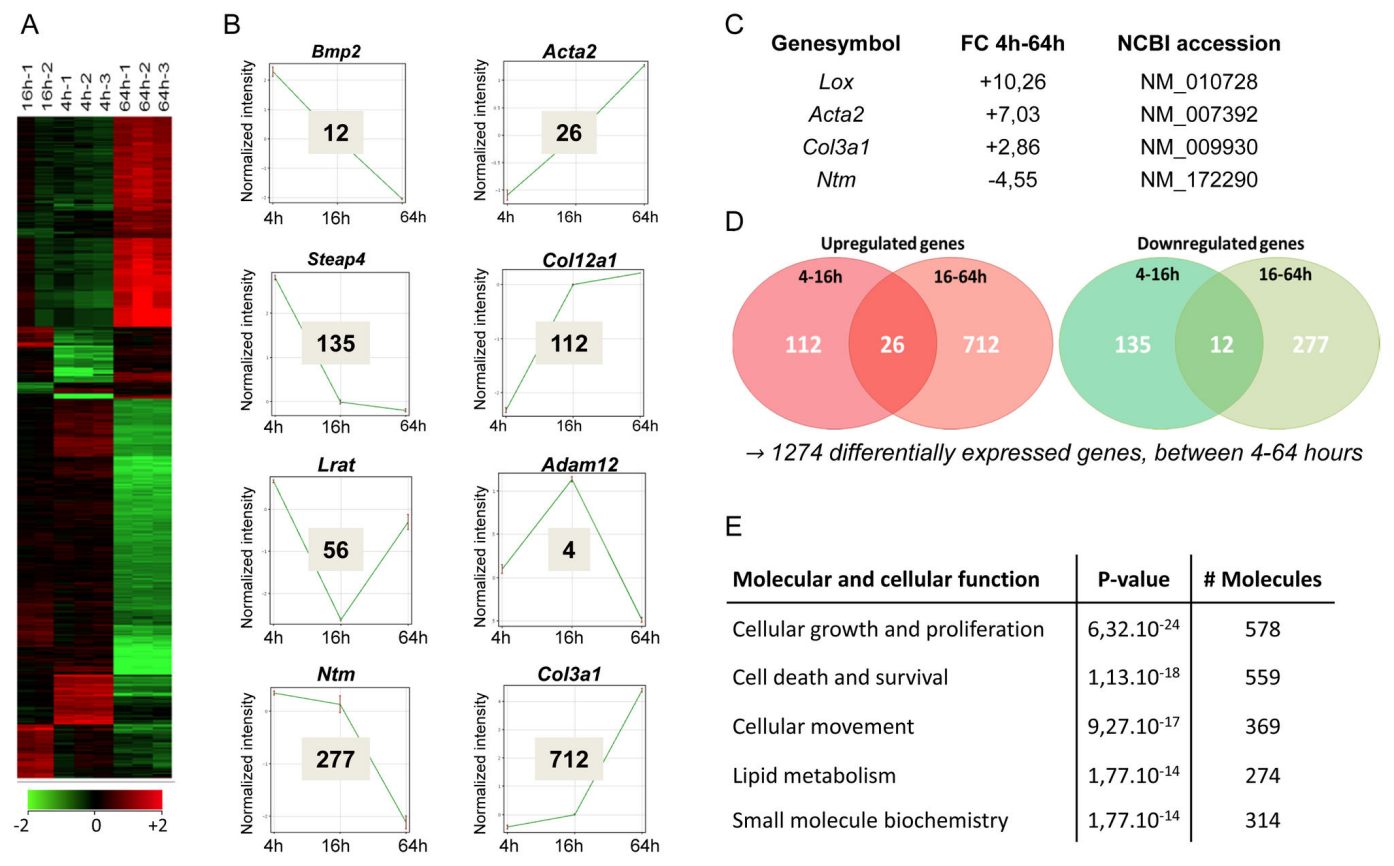
Gene expression pattern changes during early culture induced mHSC activation. Hepatic stellate cells were isolated from normal mice and cultured in 10% fetal bovine serum. 4 hours, 16 hours and 64 hours after washing of the cells mRNA was extracted, cRNA was generated and a microarray analysis was performed. (**A**) Shown is a representative graph of 2 or 3 separate HSC isolations per group (“1,” “2,” and “3”) containing all genes that were more than 2-fold up regulated (shown in red) or down regulated (shown in green) in at least 1 of the time points in comparison with quiescent HSCs (4h) (one-way anova, P < 0.05 followed by Benjamini-Hochberg correction). (**B**) Graphic representation of observed trends in gene expression changes during early HSC activation. Normalized intensities are shown and a known HSC associated gene is given that follows the trend. A representative gene for each trend is shown above each graph. (**C**) Table representing fold changes of known HSC activation/quiescence markers. (**D**) The Venn diagram shows overlapping patterns of probe sets that were significantly (P < 0.05) and at least 2-fold up regulated and down regulated between 2 time points. Probe sets that were 2-fold up regulated or down regulated in one group of which the trend was continued with a 2-fold up or down regulation between 16-64h are in the intersection of the Venn diagram. (**E**) Top 5 “molecular and cellular functions” identified by Ingenuity Pathway Analysis performed with the genes from the Venn diagram intersection.

Hierarchical clustering of genes that were at least 2-fold changed during culture induced HSC activation showed a close relation between repeats in our microarray analysis, confirming reproducibility of the isolation and culture procedures (Figure 2A). In total, 1274 genes were at least two fold changed between 4 and 64 hours in culture. Among them, well-known genes associated with HSC activation like *Acta2, Lox* and *Col3a1* were strongly up regulated while genes related to HSC quiescence such as *Ntm* [5] were down regulated between 4 and 64 hours of culture (Figure 2C). Further analysis revealed eight different trends in gene expression patterns in which known HSC-related genes are represented (Figure 2B). Only a minority of the genes changed between 4 and 16 hours of culture. Most alterations occurred between 16 and 64 hours (Figure 2D). Of the early changed genes, 38 continued the trend observed between 4 and 16 hours towards 64 hours (top 2 panels in Figure 2B). Among them were *Acta2, Col5a2, Col8a1* which were up regulated, and *Bmp2, Cxcl1* and *Cxcl2* that were down regulated. Top 30 lists of the changes between the different time points are available in Table S1. Ingenuity pathway analysis of the progressively down and up regulated genes showed that most of these genes are involved in “cellular growth and proliferation”, “cellular movement” and “lipid metabolism” (Figure 2E). These processes are all known to be linked to HSC activation and shows that the fibrogenic program is activated quickly after seeding of freshly isolated HSCs.

### Valproic acid treatment significantly changes the gene expression pattern of hepatic stellate cells

Since VPA treatment can at least partially prevent and reverse HSC activation [13], we included VPA treated HSCs in the gene expression profiling. Of the genes changed during HSC activation, the expression of 147 genes was normalized by VPA treatment to levels similar to the 4 hours control expression level. 

**Figure 3:**
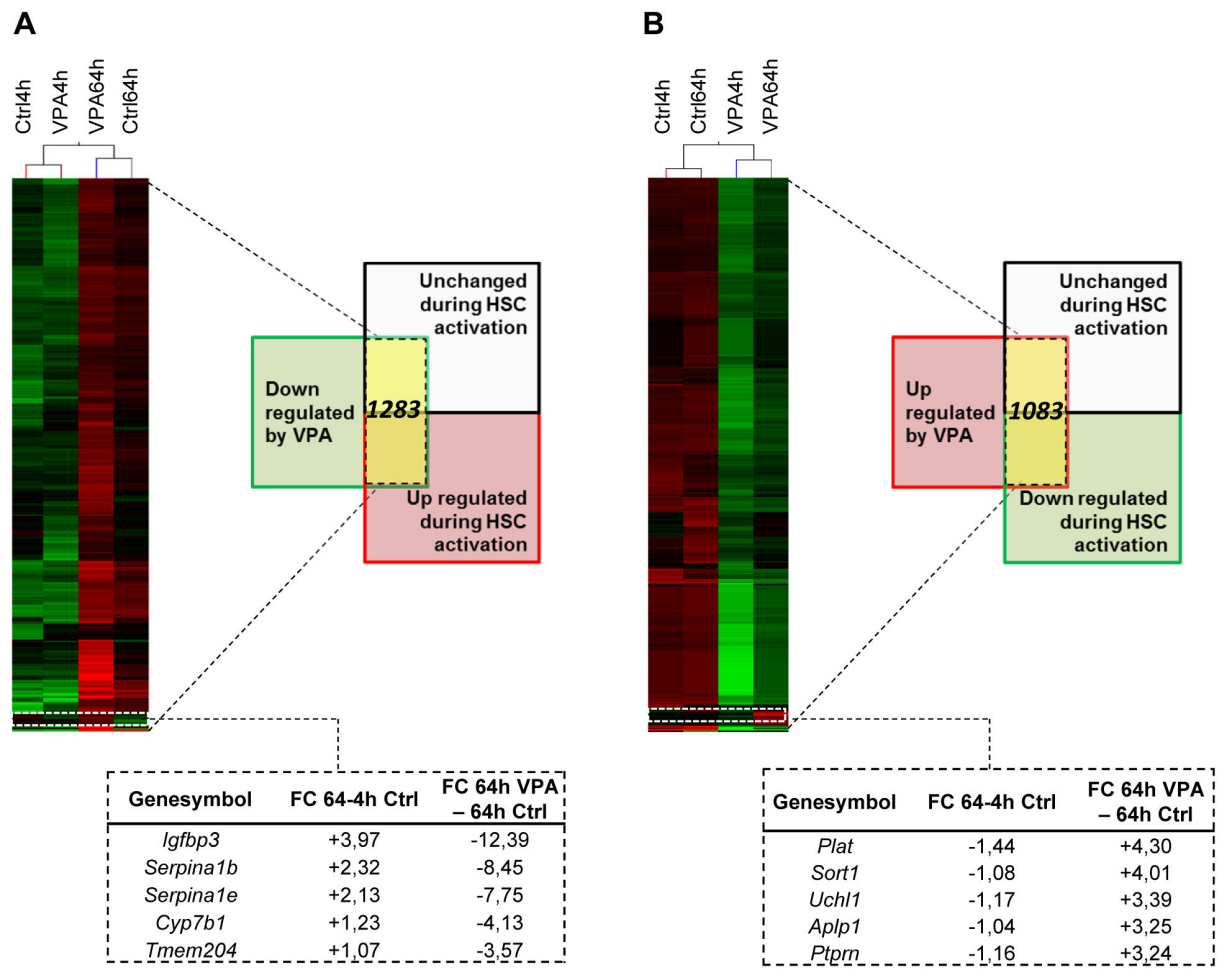
Gene expression changes by VPA treatment. (**A**) Hierarchical clustering analysis on genes with statistical significance (one-way anova, P < 0.05 followed by Benjamini-Hochberg correction), with at least a 2-fold down regulation by valproic acid (VPA) treatment (Ctrl64h vs. VPA64h) and with an up regulated/unchanged expression during hepatic stellate cell (HSC) activation. (**B**) Hierarchical clustering analysis on genes with statistically significance (one-way anova, P < 0.05 followed by Benjamini-Hochberg correction), with at least a 2-fold up regulation by VPA treatment (Ctrl64h vs. VPA64h) and with a down regulated/unchanged expression during HSC activation. Gene selection used for clustering analysis is highlighted in yellow in both schemes and the 5 top genes from this group are extracted from the clustering tables. Green color indicates a low expression value, black color indicates an intermediate expression value and red color indicates a high expression value.

Clustering analysis was used to identify prevalent gene expression patterns. Only statistically significant differentially expressed genes after 64 hours of VPA treatment (vs. 64 hours Control) were considered in the clustering algorithm, since we were interested in genes that were modified when HSC activation was inhibited. Using this approach, we selected two gene expression patterns. On the one hand, we grouped 1283 genes that were unchanged or up regulated during HSC activation and at least 2-fold down regulated after VPA treatment (Figure 3A) and on the other hand we assembled 1083 genes with unchanged or down regulated expression during HSC activation and at least 2-fold up regulated expression after VPA treatment (Figure 3B). The top-5 of these two predefined groups was further investigated in *in vitro* and *in vivo* activated cells, and in different freshly isolated liver cell types. A more elaborate list of VPA sensitive genes is shown in Table 2A. Noteworthy is that VPA-dependent up regulation of genes mostly occurs in genes that are not differentially expressed during HSC activation (Figure 3B table).

### Identification of Igfbp3 as a novel gene associated with hepatic stellate cell activation

**Figure 4:**
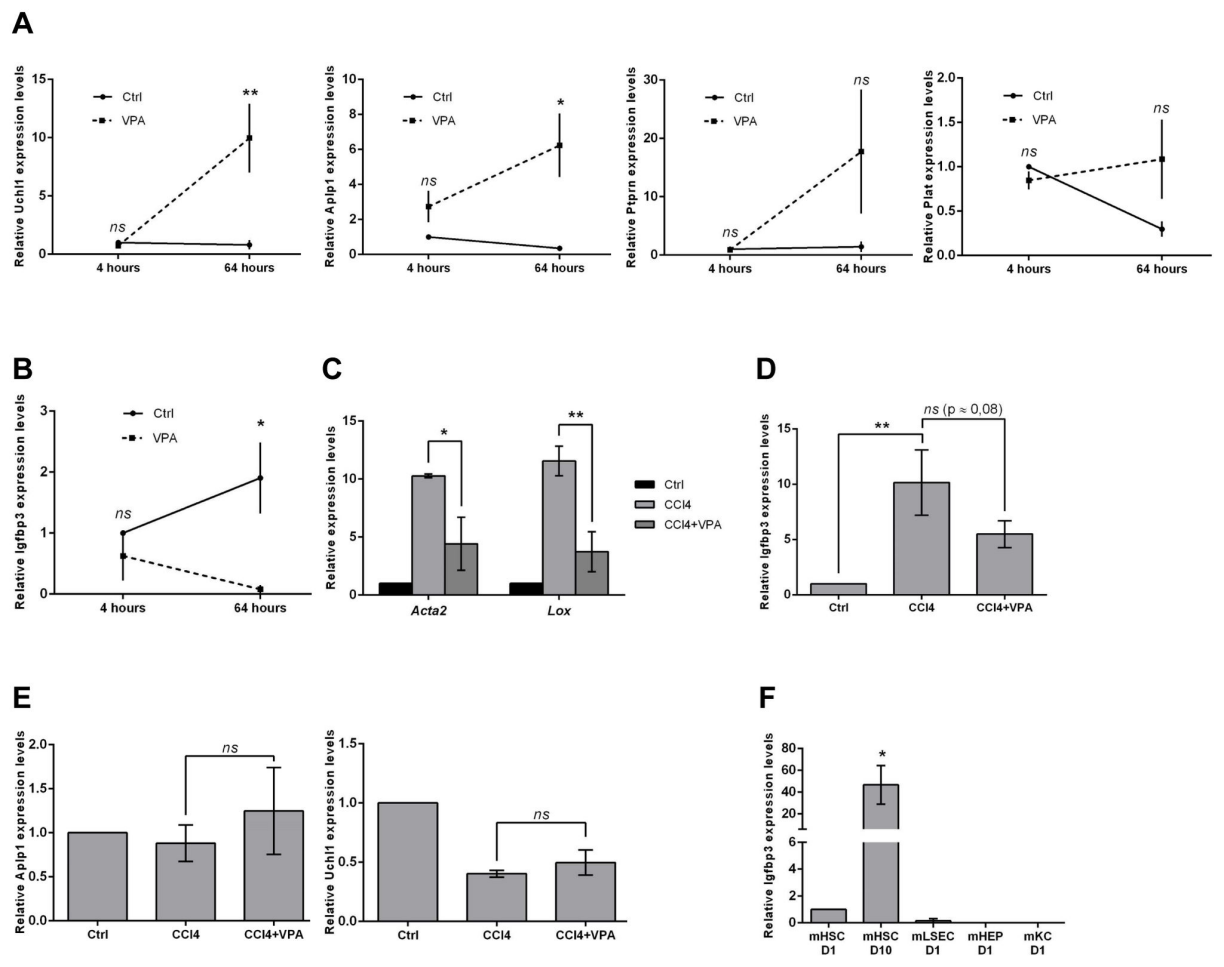
Validation of VPA-dependent genes during HSC activation *in* *vitro* and *in* *vivo*. (**A**, **B**) mRNA levels of *Uchl1*, *Aplp1*, *Prtpn, Plat* and *Igfbp3* in *in* *vitro* activating HSCs cultured for 4 or 64 hours with or without VPA supplementation determined by qPCR. n=3 (**C**) mRNA expression of HSC activation markers *Acta2* and Lox in HSCs isolated from CCl_4_ treated mice , (**D**) *Uchl1*, *Aplp1* and *Igfbp3* mRNA levels in *in* *vivo* activated hepatic stellate cells (HSCs). The cells were isolated from Balb/C jicco CBY mice that were treated for 2 weeks with CCl_4_ with or without VPA supplementation to the drinking water. (**E**) Different liver cell types were isolated from healthy mouse livers; hepatocytes were obtained with Percoll gradients, HSCs with Nycodenz gradients and KC and LSECs were isolated using respectively FACS based F4/80- and CD146-FITC-positivity. QPCR analysis was performed for *Igfbp3* mRNA in the different liver cell types. Experiments were repeated at least 2 times. In the graphs, the results are displayed as means ± SEM. ns = not significant p ≥ 0.05, * p < 0.05, ** p < 0.01.

To confirm the top-5 genes from of our profiling analysis, qPCR was performed on biological repeats of freshly isolated HSCs cultured with or without VPA supplementation. Within the group of VPA-up regulated genes, Ubiquitin carboxy-terminal hydrolase L1 (*Uchl1*), amyloid beta (A4) precursor-like protein 1 (*Aplp1*), plasminogen activator tissue (*Plat*) and protein tyrosine phosphatase receptor type N (*Ptprn*) were constantly expressed or down regulated during early activation and clearly up regulated after 64 hours VPA treatment, confirming the profiling results (Figure 4A). With respect to the top VPA-down regulated genes, only *Igfbp3* profiles were confirmed by qPCR analysis (Figure 4B). The other VPA-down regulated genes identified by the expression profiling could not be confirmed as significantly changed genes by VPA treatment (results not shown). To get insight into the *in vivo* relevance of the observed gene expression alterations, we investigated the *in vitro* validated genes in HSCs that were isolated from mice treated with CCl_4_ ± VPA for 2 weeks. In such VPA-treated mice, HSCs are less activated [18] as shown by the expression levels of *Acta2* and *Lox* in HSCs isolated from these mice (Figure 4C). We observed an up regulation of *Igfbp3* following CCl_4_ treatment while this increase was reduced in mice treated with CCl_4_ and VPA (Figure 4D). On the other hand for *Uchl1, Aplp1, Ptprn* and *Plat*, the VPA-dependence of these genes *in vivo* did not match with the *in vitro* mRNA changes (Figure 4E and results not shown). To determine whether *Igfbp3* is expressed in other liver cell types besides HSCs, *Igfbp3* mRNA levels were investigated in freshly isolated liver cell types. The highest *Igfbp3* expression was observed in 10 day-activated HSCs when compared to quiescent HSCs, hepatocytes, Kupffer cells and liver sinusoidal endothelial cells (Figure 4F). The early up regulation of *Igfbp3* expression in HSCs observed at 64 hours clearly continues to levels that reach an approximately 50-fold induction at day 10. Together, these data suggest that *Igfbp3* is a marker of activated HSCs.

### Igfbp3 is important for the stellate cell migratory capacity during activation

**Figure 5:**
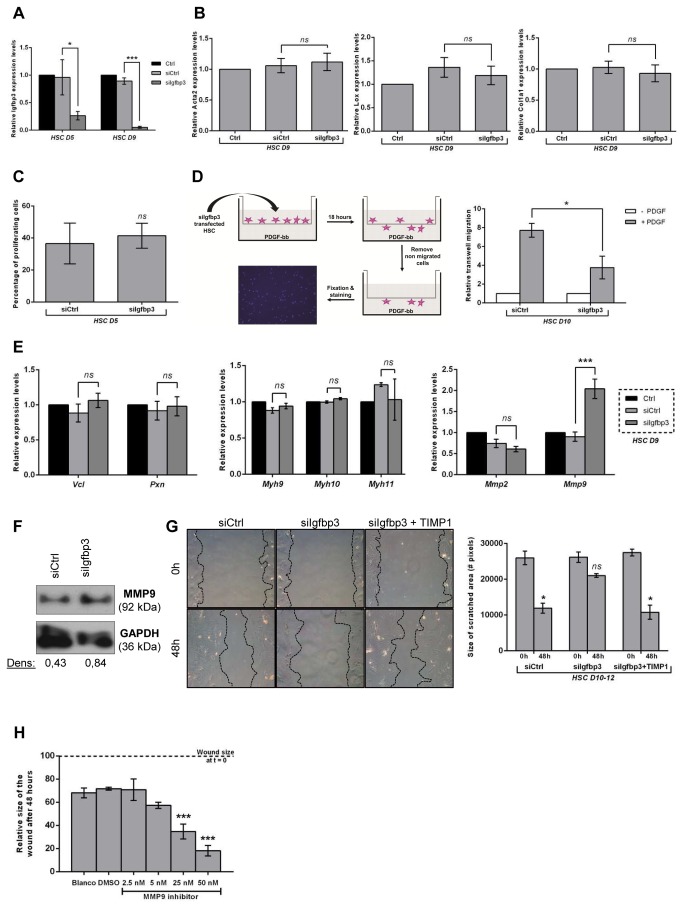
Effect of Igfbp3 knock-down on gene expression, migration and proliferation in activating HSCs (A) mRNA levels of *Igfbp3* in activating day 5 HSCs (HSC D5, once transfected at day 1) and day 9 HSCs (HSC D9, twice transfected at day 5/day7) transfected with a control siRNA (siCtrl) or with an siRNA for *Igfbp3* (siIgfbp3). (**B**) *Acta2*, Lox and *Col1a1* mRNA levels in activated day 9 HSCs transfected with siCtrl or with siIgfbp3 (**C**) Influence of *Igfbp3* knock-down on the proliferation of hepatic stellate cells (HSCs). At day 2, siIgfbp3-transfected (at day 1) and siCtrl-transfected HSCs were exposed to EdU, fixed 2 days later and stained for DNA-incorporated EdU. The percentage of EdU-positive cells is given. (**D**) Principle of PDGFbb-induced transwell migration (left). siIgfbp3 transfected HSCs were plated at day 9 in transwell inserts. Migration was determined 18 hours (h) later and compared to HSCs transfected with siCtrl. Results are relative to siCtrl/siIgfbp3 transfected HSCs without PDGFbb supplementation (right). (**E**) Vinculin (Vcl), Paxillin (Pxn), myosin heavy chain 9, 10, 11 (Myh9, -10, -11) and matrix metalloproteinase 2, 9 (Mmp2, -9) mRNA levels in activated day 9 HSCs transfected with siCtrl or with siIgfbp3 (**F**) Protein levels for MMP9 and GAPDH on day 9 HSCs transfected twice with siCtrl or siIgfbp3. Densitometry values (dens) for MMP9 relative to the GAPDH loading control are displayed below the Western blot. (**G**) Representative images of HSCs at 0 and 48h after wounding (left), and quantification of migration (right). HSCs were transfected with siCtrl, siIgfbp3 or siIgfbp3 in combination with a supplementation of 100 ng/mL recombinant murine TIMP-1 (siIgfbp3 + TIMP-1). Experiments were repeated at least 3 times. Results in the graphs are expressed as means ± SEM. (**H**) The contribution of MMP9 to HSC migration was evaluated by treatment of activated HSCs with an MMP9 selective inhibitor, using different concentrations, n=2. Migration is expressed as relative to the width of the wound at the start of the scratch. ns = non-significant p ≥ 0.05, * p < 0.05, ** p < 0.01, *** p < 0,001.

To evaluate the importance of *Igfbp3* during HSC activation, the gene was silenced in freshly isolated HSCs by siRNA transfection. QPCR on day 5 and day 9 demonstrates a successful knock-down (Figure 5A). Despite this knock-down, expression of HSC activation markers *Acta2*, *Lox* and *Col1a1* (Figure 5B) did not change significantly and proliferation was not affected (Figure 5C). Functionally, Igfbp3 knock-down reduced migration of day 9 activated HSCs in a PDGFBB dependent transwell assay (Figure 5D). Prompted by the transwell assay results, we investigated the expression of migration related genes in *Igfbp3* knock-down cells. We looked at cell-ECM adhesion molecules paxillin (*Pxn*) [19] and vinculin (*Vcl*) [20], cell migration and contraction related myosins (myosin heavy chain II A and B; *Myh9* and *Myh10* resp and Smooth muscle myosin, *myh11*)[21] and at the expression of matrix metalloproteinases (*Mmp2* and *Mmp9*). Of the tested genes only the expression of MMP9 was influenced by *Igfbp3* silencing and the up regulation of MMP9 could also be confirmed on protein level by WB (Figure 5E, F). To test whether the deregulation of MMP9 could explain the reduced migration, we performed a rescue experiment using recombinant murine TIMP-1 in the wound healing assay. 100 ng/mL TIMP-1 administration restored the migratory capacity of the siIgfbp3-transfected HSCs in the wound healing assay (Figure 5G). To further confirm that these observations are related to MMP9 regulation; the mRNA expression of *MMP1a, MMP3, MMP11, MMP13* and *MMP19* was analyzed. These were all unaffected by silencing of Igfbp3 (Figure S1). To test the involvement of MMP9 in HSC migration we have used an MMP9 selective inhibitor. Treatment with this compound lead to a dose-dependent decrease of the wound size in the wound healing experiments. (Figure 5H).

## Discussion

In their quiescent state, HSCs are responsible for maintaining a normal ECM turnover. This process is tightly regulated by the production of matrix proteins and matrix remodeling enzymes. Different stimuli like viruses, alcohol, reactive oxygen species, and adipocytokines can lead to activation of HSCs that produce abnormal amounts of ECM and inflammatory mediators, leading to liver fibrosis. Although, the activation of HSCs has been extensively studied, very little information is available on the changes at gene expression level during the onset of myofibroblastic differentiation, as most profiling studies have been performed on cells cultured for at least 24 hours [5,11,12]. In contrast, in this study we focused on finding key genes that are important during early HSC activation (4h, 16h and 64h). In addition, we chose to compare expression profiles of normal HSC cultures, with profiles of HSCs treated with VPA that can maintain HSCs in a quiescence-like state [13]. We hypothesised that this inhibitor would preserve quiescence by altering key regulators of the activation process thereby facilitating the identification of such genes. 

Our gene expression profiling approach allowed the identification of genes changing between 4 and 16 hours in culture. We observed 8 different expression trends during early stellate cell activation. Of the 1274 genes with altered expression, only a minority of the genes changed progressively between the 3 time points (12 down regulated and 26 up regulated). This emphasizes the importance of implying multiple time points, when conclusions on gene expression changes are made. In four of the trends the expression was stable (<2 fold, represented by *Steap4, Col12a1, Ntm* and *Col3a1*) between 2 of the 3 analyzed time points. A pathway analysis was performed for these genes showing that steroid biosynthesis and cell cycle pathways genes were enriched in the up regulated genes while complement and coagulation cascade pathway genes were enriched in the down regulated genes (Table S3). For 60 genes there was a transient change between 4-16-64 hours in culture. Although potentially interesting, these transient changes are difficult to interpret. They could be true initiation steps of the activation process, but the functional analysis of these changes by e.g. siRNAs or overexpression is difficult to perform due to the transient nature of their transcriptional regulation in culture. We therefore focused on those genes that showed a clear trend between 4, 16 and 64 hours in culture and that were influenced by VPA (Figure 3).

Gene expression profiling lead to the identification of 2366 genes that were either up regulated/unchanged during early HSC activation and down regulated by VPA or *vice versa* (Figure 3). Inhibition of HDAC activity in general leads to hyper acetylation, a permissive chromatin structure and consequently increased transcription [22]. Genes in our array following this assumption are *Uchl1*, *Aplp1*, *Ptprn* and *Plat*. All four of them have been reported to have distinct functions in neuronal cells, from ubiquitin system protein, to glucose uptake regulator or initiator of ECM remodeling [23–28]. This makes them potentially interesting genes to study in HSCs, but the lack of robustness between our *in vitro* and *in vivo* results made us exclude them from initial functional tests. 

Some of our identified VPA-sensitive genes, like *Uchl1*, *sortilin 1* (*Sort1*), *regulator of chromosome condensation and BTB domain containing* protein *2* (*Rcbtb2*), *collagen,* type *III,* alpha *1* (*Col3a1*) and *Emilin1* (Figure 4A and Table S1D) were also regulated by HDAC inhibitors in other studies, suggesting that the effects of HDAC-inhibition are conserved between different cell model systems and species [29,30]. For other genes, such as *Tmem204* and *Cyp7b1*, qPCR analysis did not confirm the observations from the gene profiling analysis. These variabilities could be due to the differences in methodologies used for microarray and qPCR. One example is the normalization, which is a global normalization of the microarray data, while for qPCR analysis gene expression of all genes is corrected with the expression of the housekeeping gene GAPDH, known to perform well in HSC *in vitro* cultures. Of our VPA affected genes, most were changed only at later time points and thus we did not identify new early key regulators as we had set out at the start of our study. This could be due to the use of VPA as a tool to identify or rather focus on genes that are modified when a more quiescent phenotype is maintained *in vitro*. Furthermore, our criteria that a gene identified by our approach during culture-induced activation of HSCs should also be regulated in a similar manner during *in vivo* activation in the CCl_4_ mouse model, limited most likely the amount of positive hits.

Despite this, we could reproducibly confirm changes in *Igfbp3* expression in *in vitro* activated HSCs and in cells isolated from mice treated with CCl_4_ and mice that received CCl_4_ and VPA for 2 weeks. This gene is a member of the family of insulin-like growth factor binding proteins and is able to modulate the activity of insulin-like growth factors by forming a ternary complex with insulin-like growth factor acid-labile subunit (IGFALS) and either IGF1 or IGF2. IGFBP3 can either stimulate or inhibit the growth promoting effects of the IGFs by presenting or sequestering IGFs to/from their receptor [31,32]. Of the different IGFBPs, it is found that IGFBP3 is responsible for binding of 70–90% of all circulating IGF1. As IGF1 is a cytokine with differential effects on quiescent and activated stellate cells [33,34], these changes could be related to alterations in IGFBP3 levels. De Minicis et al. described *Igfbp3* to be strongly up regulated in *in vitro* activated HSC samples (see supplemental information in [5]) while Boers et al. demonstrated the up regulation of multiple IGFBP proteins in activated human HSCs [35]. Both studies did not further investigate IGFBP3 and its role during HSC activation. Our data confirms an early study by Gentilini et al. showing a TGFβ dependent up regulation of *Igfbp3* in human cells isolated from liver wedges and an increase of *Igfbp3* mRNA expression in the livers of cirrhotic patients [36]. We show that *Igfbp3* is almost exclusively expressed in activated HSCs when compared to other liver cell types and that it is highly expressed in freshly isolated HSCs after *in vivo* activation. Many intracellular and extracellular functions have been described for IGFBP3 depending on the studied model and microenvironment. Its functions range from “simple” IGF1 regulator to modulator of gene transcription [37], autophagy regulator [38], and inducer of the TGFβ receptor system [39,40]. While in the Gentilini study, the focus was mainly on the extracellular roles as they used recombinant IGFBP3 treatment, in this study we have silenced *Igfbp3* using siRNAs. This approach not only affects the extracellular but also the intracellular levels of IGFBP3 and allowed us to investigate the effects on cell proliferation and migration independent of IGF1. Most of the tested activation markers such as *Acta2, Lox*, and *Col1a1* were not affected by *Igfbp3* knockdown, also no changes in cell proliferation and no obvious cell death were observed after silencing. Features of HSC activation that were significantly altered by *Igfbp3* silencing were *Mmp9*/MMP9 expression and cell migration (Figure 5). Several studies have described the *in vitro* migration potential of hepatic stellate cells; mostly in response to chemoattractants [7,41–44]. However, proof for the *in vivo* contribution of this process to fibrogenesis is limited to histological observations [45,46]. It is assumed that the accumulation of α-SMA positive, desmin positive cells in areas of tissue injury and resulting in fibrotic septa is the consequence of local proliferation and relocation of HSCs [47]. In our hands, only MMP9 and not the expression of other migration related genes such as vinculin, paxillin and myosins was influenced by Igfbp3 knockdown.

The regulation of cell attachment or release of matrix proteins is complex and may influence cell motility and migration. How ECM is linked to cell migration is still under debate. Commonly, the ECM is considered as a barrier for cell migration and excessive degradation of matrix proteins by MMPs could thus free the path for cell migration. However, in our study we observe a clear reduction of HSC migration when cells express higher levels of *Mmp9*/MMP9 after *Igfbp3* knock-down and this in both transwell and wound healing assays. Analogous observations were described by others [48–50] and a recent paper by Xue et al. suggested that high MMP9 levels lead to excessive degradation of matrix proteins, which might be desirable to support and direct cell migration [51–54]. MMP9 might also cleave other non-matrix proteins essential for cell migration [48]. In addition, MMP-induced cleavage of IGFBP3 contributes to IGF1 induced cell adhesion [55] which is in line with our results. To strengthen our hypothesis that the observed effects on cell migration after Igfbp3 silencing are related to the Mmp9 increase, we have performed a rescue experiment using recombinant TIMP1, a well-known endogenous inhibitor of MMP2/9. This resulted in wound closure even after Igfbp3 knock down, suggesting that the Mmp9 up regulation is an important factor in the regulation of HSC migration. Timp1 preferentially binds to Mmp9 and to lesser extend to Mmp3 [56] and indeed we could specifically show the contribution of MMP9 to mHSC migration by treating the cells with an MMP9 selective inhibitor.

Noteworthy, we show a reduced expression of Igfbp3 following VPA treatment while the HDAC inhibitors sodium butyrate and trichostatin A have been shown to stimulate transcription of the *Igfbp3* gene in a variety of cell lines, including liver, breast, colon, and prostatic epithelial cells [57–60]. Furthermore, the studies reporting an up regulation of *Igfbp3* following HDAC inhibition, show that this is due to hyperacetylation of the Igfbp3 promoter [57–60]. The pronounced down regulation of *Igfbp3* in our experimental setting is therefore likely to be a strong, but indirect consequence of the VPA treatment. At this moment the identities of repressors or microRNAs that could direct this VPA-mediated down regulation remain unknown.

In patients with advanced liver injury, caused by hepatitis C infection or steatosis, serum levels of IGFBP3 are increased [61,62] which could be an indication of HSC activation. Only livers from severely cirrhotic patients showed increased levels of Igfbp3 mRNA [36], a state which could not be established in our 2-6 weeks CCl_4_-treated animals (data not shown). Selective *in vivo* modulation of IGFBP3 could perhaps prevent the evolution of mild to more severe liver fibrosis.

We conclude from this study that *Igfbp3* is a marker of culture-induced HSC activation, but that it plays no significant role in the regulation of activation or proliferation of HSCs. Nevertheless, IGFBP3 has a function in HSC migration and possibly wound repair.

## Supporting Information

Table S1 **Top list of genes changed during the early time points of mHSC activation.** mHSCs were freshly isolated and cultured for 4, 16 and 64 hours after washing. At these timepoints cells were collected for RNA analysis, using the Affymetrix GeneChip Mouse Gene 1.0 ST array. This table gives an overview of the fold changes for the 30 most down and 30 most up regulated genes between 4-16 hours and 16-64 hours in culture. The right table shows genes of which the expression is changed at least two fold between 4-16 hours and again more than two fold between 16-64 hours.
(DOCX)

Table S2 **Differentially expressed genes after 64 hours VPA treatment.** The top-30 up regulated (left) or down regulated (right) genes during 64 hours VPA treatment (Ctrl64h versus VPA64h, one-way ANOVA, p < 0,05).
(DOCX)

Table S3 **Pathway analysis on selected gene expression trends.** A DAVID Enrichment analysis was performed for the genes from the selected trends. Default setting were used. Term: Gene set name; Count: number of genes associated with this gene set; Percentage: gene associated with this gene set/total number of query genes; P-value: modified Fisher Exact P-value; Fold enrichment: measures the magnitude of enrichment in the input gene list compared to a background set; Bonferroni: P-value after multiple testing corrections.
(DOCX)

Figure S1 **Expression of *MMPs* in mHSCs after *Igfbp3* silencing.** (**A**) mRNA levels of *MMPs* were investigated by QPCR in day 9 HSCs (HSC D9, twice transfected at day 5/day7) transfected with a control siRNA (siCtrl) or with an siRNA targeting *Igfbp3* (siIgfbp3).
(DOCX)
