# Supplementary material for: Gene Expression Profiling of Early Hepatic Stellate Cell Activation Reveals a Role for Igfbp3 in Cell Migration
Source: PLoS One. 2013 Dec 17;8(12):e84071. doi: 10.1371/journal.pone.0084071 (PMC3866247; doi:10.1371/journal.pone.0084071)
Supplement: Table S1 — Top list of genes changed during the early time points of mHSC activation. mHSCs were freshly isolated and cultured for 4, 16 and 64 hours after washing. At these timepoints cells were collected for RNA analysis, using the Affymetrix GeneChip Mouse Gene 1.0 ST array. This table gives an overview of the fold changes for the 30 most down and 30 most up regulated genes between 4-16 hours and 16-64 hours in culture. The right table shows genes of which the expression is changed at least two fold between 4-16 hours and again more than two fold between 16-64 hours. (DOCX) [file pone.0084071.s001.docx]

**Table S1. Top list of genes changed during the early time points of mHSC activation.**
