# Supplementary figures and images for: Gene Expression Profiling of Early Hepatic Stellate Cell Activation Reveals a Role for Igfbp3 in Cell Migration
Source: PLoS One. 2013 Dec 17;8(12):e84071. doi: 10.1371/journal.pone.0084071 (PMC3866247; doi:10.1371/journal.pone.0084071)

**Table S2. Differentially expressed genes after 64 hours VPA treatment.**


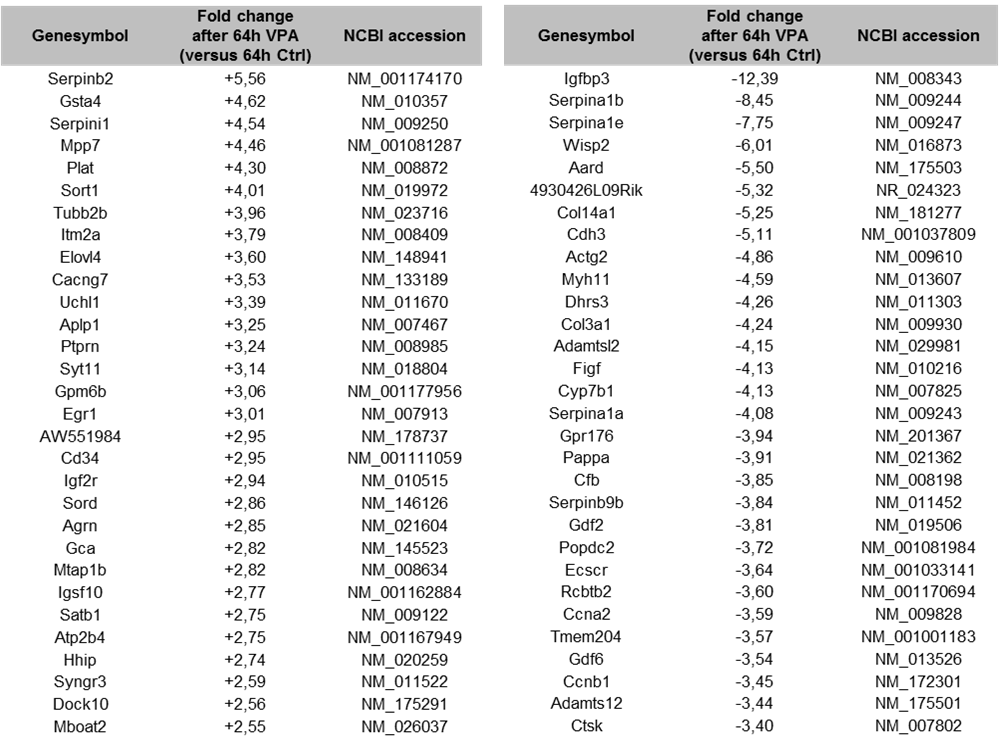

Supplement: Table S2 — Differentially expressed genes after 64 hours VPA treatment. The top-30 up regulated (left) or down regulated (right) genes during 64 hours VPA treatment (Ctrl64h versus VPA64h, one-way ANOVA, p < 0,05). (DOCX) [file pone.0084071.s002.docx]

**Figure S1.**


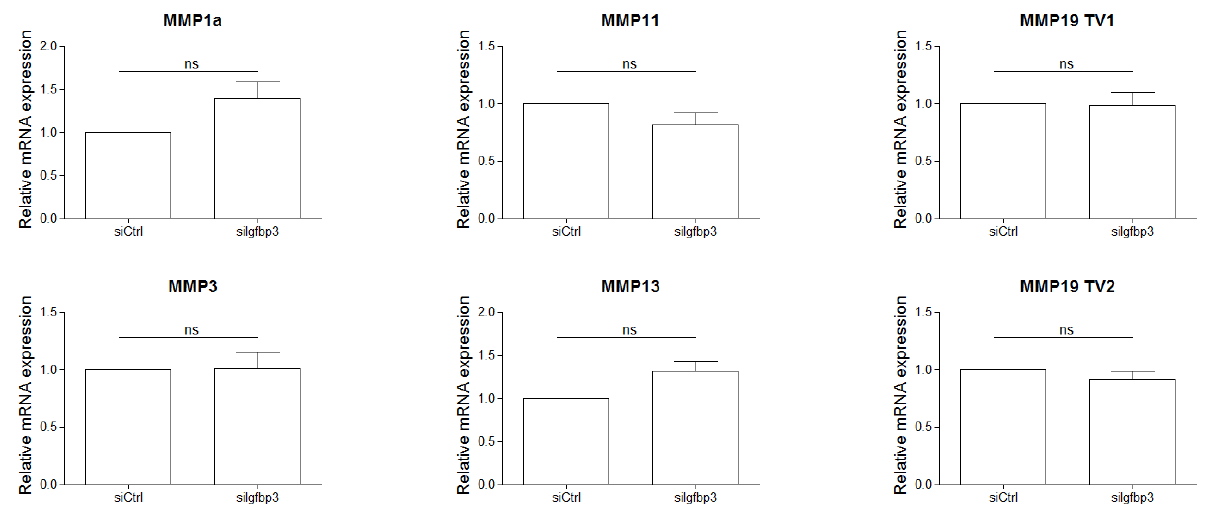

Supplement: Figure S1 — Expression of MMPs in mHSCs after Igfbp3 silencing. (A) mRNA levels of MMPs were investigated by QPCR in day 9 HSCs (HSC D9, twice transfected at day 5/day7) transfected with a control siRNA (siCtrl) or with an siRNA targeting Igfbp3 (siIgfbp3). (DOCX) [file pone.0084071.s004.docx]
