# Supplementary material for: Gene Expression Profiling of Early Hepatic Stellate Cell Activation Reveals a Role for Igfbp3 in Cell Migration
Source: PLoS One. 2013 Dec 17;8(12):e84071. doi: 10.1371/journal.pone.0084071 (PMC3866247; doi:10.1371/journal.pone.0084071)
Supplement: Table S3 — Pathway analysis on selected gene expression trends. A DAVID Enrichment analysis was performed for the genes from the selected trends. Default setting were used. Term: Gene set name; Count: number of genes associated with this gene set; Percentage: gene associated with this gene set/total number of query genes; P-value: modified Fisher Exact P-value; Fold enrichment: measures the magnitude of enrichment in the input gene list compared to a background set; Bonferroni: P-value after multiple testing corrections. (DOCX) [file pone.0084071.s003.docx]

**Table S3. Pathway analysis on selected gene expression trends.**
